# FOLFOXIRI (folinic acid, 5-fluorouracil, oxaliplatin and irinotecan) *vs* FOLFIRI (folinic acid, 5-fluorouracil and irinotecan) as first-line treatment in metastatic colorectal cancer (MCC): a multicentre randomised phase III trial from the Hellenic Oncology Research Group (HORG)

**DOI:** 10.1038/sj.bjc.6603011

**Published:** 2006-02-28

**Authors:** J Souglakos, N Androulakis, K Syrigos, A Polyzos, N Ziras, A Athanasiadis, S Kakolyris, S Tsousis, Ch Kouroussis, L Vamvakas, A Kalykaki, G Samonis, D Mavroudis, V Georgoulias

**Affiliations:** 1 Department of Medical Oncology, University General Hospital of Heraklion, PO Box 1352, Heraklion, Crete 71110, Greece; 2 Third Department of Internal Medicine, School of Medicine, University of Athens, ‘Sotiria’ General Hospital of Athens, Greece; 3 Department of Propedeutic Medicine, Medical Oncology Unit, School of Medicine, University of Athens, ‘Laikon’ General Hospital of Athens, Greece; 4 First Department of Medical Oncology, ‘METAXA'S’ Anticancer Hospital of Athens, Greece; 5 Department of Medical Oncology, General Hospital of Larissa, Greece; 6 Department of Medical Oncology, University General Hospital of Alexandroupoli, Greece; 7 Department of Medical Oncology, ‘Venizelion’ General Hospital of Heraklion, Greece; 8 First Department of Medical Oncology, ‘Theagenion’ Anticancer Hospital of Thessaloniki, Greece

**Keywords:** FOLFOXIRI, metastatic, colorectal cancer

## Abstract

To compare the efficacy and toxicity of oxaliplatin (L-OHP) in combination with irinotecan (CPT-11), 5-fluorouracil (5-FU) and leucovorin (LV) (FOLFOXIRI) *vs* irinotecan and 5-FU/LV (FOLFIRI) as first-line treatment of patients with metastatic colorectal cancer (MCC). A total of 283 chemotherapy-naïve patients with MCC were enrolled (FOLFIRI arm: *n*=146; FOLFOXIRI arm: *n*=137). In the FOLFOXIRI arm, CPT-11 (150 mg m^−2^) was given on d_1_, L-OHP (65 mg m^−2^) on d_2_, LV (200 mg m^−2^) on days 2 and 3 and 5-FU (400 mg m^−2^ as i.v. bolus and 600 mg m^−2^ as 22 h i.v. continuous infusion) on days 2 and 3. In the FOLFIRI arm, CPT-11 (180 mg m^−2^) was given on d_1_ whereas LV and 5-FU were administered in the same way as in the FOLFOXIRI regimen. Both regimens were administered every 2 weeks. There was no difference in terms of overall survival (median OS: 19.5 and 21.5 months, for FOLFIRI and FOLFOXIRI, respectively; *P*=0.337), median time to disease progression (FOLFIRI: 6.9 and FOLFOXIRI: 8.4 months; *P*=0.17), response rates (33.6 and 43% for FOLFIRI and FOLFOXIRI, respectively; *P*=0.168). Patients treated with FOLFOXIRI had a significantly higher incidence of alopecia (*P*=0.0001), diarrhoea (*P*=0.0001) and neurosensory toxicity (*P*=0.001) compared with patients treated with FOLFIRI. The present study failed to demonstrate any superiority of the FOLFOXIRI combination compared with the FOLFIRI regimen, although the observed median OS is one of the best ever reported in the literature.

Colorectal cancer (CRC) is the second most common cancer in Europe (Ferlay *et al*, 1999), accounting for 8% of all malignant tumours in adults (Parkin *et al*, 2005), and the third in the USA, with approximately 145 000 new cases expected in 2005 (American Cancer Society, 2005). Despite a macroscopically curative surgical resection in 70–80% of patients, almost half of them will develop local recurrence or/and metastatic disease and will die of the disease (Kohne *et al*, 1998).

Since 1957 and until the last decade, 5-fluorouracil (5-FU) was the only active cytotoxic agent against advanced CRC. Furthermore, there is no internationally accepted ‘standard’ 5-FU/leucovorin (LV) regimen; the monthly 5-day i.v. bolus North Central Treatment Group, ‘Mayo Clinic’ regimen, is commonly used (Poon *et al*, 1989); however, in Europe, a biweekly regimen combining bolus and infusional 5-FU modulated by LV (‘de Gramont’ schedule) is commonly used in the previous years. Recent studies have demonstrated that the de Gramont regimen was more effective and had a better safety profile than the LV/FU bolus regimen (de Gramont *et al*, 1997).

Irinotecan (CPT-11, Campto®, Pfizer Pharmaceuticals, New York, NY, USA) is a hemi-synthetic, water-soluble derivative of the plant alkaloid camptothecin. After conversion in the liver to its active metabolite, SN-38, CPT-11 acts by inhibiting the eucariotic enzyme DNA-topoisomerase I (Hsiang *et al*, 1989; Tanizawa *et al*, 1994). Phase III studies in patients with metastatic CRC (MCC) clearly demonstrated a significant survival advantage for irinotecan combined with LV/FU, compared with LV/FU alone (Douillard *et al*, 2000; Saltz *et al*, 2000). These studies established irinotecan-based chemotherapy as the ‘standard of care’ (FOLFIRI in Europe and IFL in the United States) and therefore became the preferred comparator regimens for further phase III trials.

The 1,2 diaminocyclohexane (DACH) platinate oxaliplatin (L-OHP; Eloxatin, Sanofi-Aventis Inc., Paris, France) differs from previously developed cisplatin and carboplatin in that it does not present free amino groups linked to platinum, but rather a cyclic, bulky, rigid structure. 1,2 Diaminocyclohexane platinates combine with DNA to form adducts resistant to DNA repair and replicative bypass (Raymond *et al*, 1998). While L-OHP seems to form the same type of lesions as cisplatin and at the same sites on DNA, L-OHP-induced adducts are more effective at inhibiting DNA synthesis (Rixe *et al*, 1996). Moreover, the only different resistance mechanisms between cisplatin and L-OHP are defects in mismatch repair and enhanced replicative bypass (Bleiberg, 1998). A randomised study has shown that LVFU2 in combination with L-OHP (FOLFOX4) was associated with a prolonged progression-free survival compared with LVFU2 (de Gramont *et al*, 2000). Moreover, FOLFOX 4 was associated with an improved median overall survival (OS) when compared with IFL (irinotecan in combination with LV/FU given i.v. bolus) (Goldberg *et al*, 2004).

*In vitro* models suggest schedule-dependent cytotoxic interactions for the combination of thymidylate synthase inhibitors with SN-38 and L-OHP; indeed, synergistic effects were demonstrated only when SN-38 was started 24–48 h before the L-OHP/5-FU/LV exposure in WIDR and SW620 human CRC cell lines (Fischel *et al*, 1999). The efficacy and tolerance of the FOLFOXIRI regimen has been recently reported; the observed response rates (RR) were 58.1 and 69%, the time to tumour progression (TTP) 11 and 10.4 months and the estimated OS 22.5 and 26.5 months in the Greek and the Italian phase II studies, respectively (Falcone *et al*, 2002; Souglakos *et al*, 2002).

Based on these promising results a multicentre randomised phase III study was conducted by the Gastrointestinal Committee of the Hellenic Oncology Research Group (HORG) in order to evaluate the efficacy and safety of the FOLFOXIRI regimen in comparison with the standard combination of FOLFIRI regimen as first-line treatment in patients with advanced CRC.

## PATIENTS AND METHODS

### Eligibility criteria

Patients with histologically documented and measurable metastatic adenocarcinoma of the colon or rectum were enrolled. Previous chemotherapy for metastatic disease was not allowed. Patients who had received prior adjuvant 5-FU-based chemotherapy were eligible if they had remained free of disease for at least 6 months after the completion of adjuvant therapy. Patients with operable metastatic disease were excluded from the study. Other eligibility criteria were: age ⩾18 years, performance status (ECOG) 0–2; at least one bidimensionally measurable lesion of ⩾2 cm; a life expectancy of at least 3 months; adequate haematologic parameters (absolute neutrophil count ⩾1.5 × 10^9^ l^−1^ and platelets ⩾100 × 10^9^ l^−1^); creatinine and total bilirubin ⩽1.25 times the upper limit of normal; aspartate and alanine aminotransferases ⩽3.0 times the upper limit of normal; absence of active infection or malnutrition (loss of more than 10% of body weight); absence of a second primary tumour other than nonmelanoma skin cancer or *in situ* cervical carcinoma. Patients treated with palliative radiotherapy had to have measurable metastatic disease outside the irradiation fields. Patients with severe cardiac dysfunction, liver metastases involving more than 50% of the liver parenchyma, chronic diarrhoea, or prior irradiation affecting more than 30% of the active bone marrow were excluded. The study was approved by the Ethics and Scientific Committees of each participating centre. All patients gave written informed consent in order to participate in the study.

### Chemotherapy

**Figure 1:**
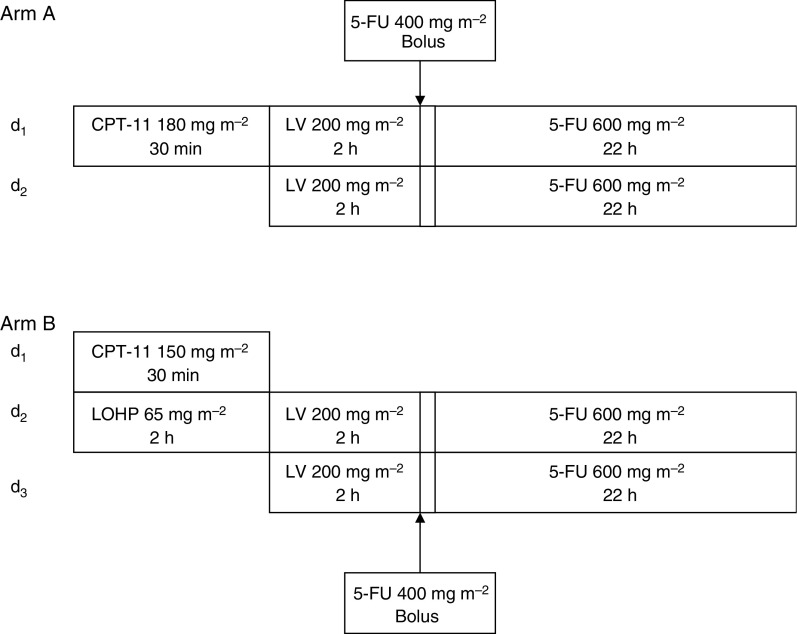
Chemotherapy regimens (**A**) FOLFIRI (folinic acid, 5-fluorouracil and irinotecan) and (**B**) FOLFOXIRI (folinic acid, 5-fluorouracil, oxaliplatin and irinotecan).

Patients were centrally randomised to receive either the FOLFIRI or the FOLFOXIRI regimen. Patients randomised in the FOLFIRI regimen (arm A) received CPT-11 at the dose of 180 mg m^−2^ as a 30 min i.vinfusion on day 1; LV was given at the dose of 200 mg m^−2^ as a 2-h i.v. infusion, followed by 5-FU 400 mg m^−2^ as i.v. bolus, and then, 600 mg m^−2^ as a 22-h continuous i.v. infusion, on days 1 and 2. Patients randomised in the FOLFOXIRI regimen (arm B) received CPT-11 at the dose of 150 mg m^−2^ as a 30 min i.v. infusion on day 1; LV was given at the dose of 200 mg m^−2^ as a 2-h i.v. infusion, followed by 5-FU 400 mg m^−2^ as i.v. bolus, and then, 600 mg m^−2^ as a 22-h continuous i.v. infusion, on days 2 and 3. Oxaliplatin was administered on day 2 at the dose of 65 mg m^−2^ as a 2-h i.v. infusion in parallel with LV but using different lines (Figure 1). Routine antiemetic prophylaxis with a 5-hydroxytryptamine-3-receptor antagonist was used in both study groups. Treatment was administered every 2 weeks until disease progression or unacceptable toxicity, or until the patient declined further treatment.

Patients were assessed for toxicity before each cycle using the National Cancer Institute Common Toxicity Criteria (National Cancer Institute, 1999). Chemotherapy was delayed until recovery if neutrophils were <1.5 × 10^9^ l^−1^ or platelets less than 100 × 10^9^ l^−1^ or for significant persisting nonhaematologic toxicity. CPT-11 was administered according to the guidelines used for CPT-11 monotherapy, including recommendations for using atropine and loperamide (Wadler *et al*, 1998). Peripheral sensory neuropathy was graded according to the L-OHP-specific scale modified from Caussanel (Caussanel *et al*, 1990): paresthaesias/dysethaesias of short duration with complete recovery before the next cycle (grade 1); paresthaesias/dysethaesias persisting between two cycles without functional impairment (grade 2); paresthaesias/dysethaesias with functional impairment (grade 3).

Doses of all drugs were reduced by 15% in subsequent cycles in case of grade 4 neutropenia or grade 3–4 thrombocytopenia lasting for more than 3 days or in case of febrile neutropenia. No prophylactic administration of granulocyte colony-stimulating factor (G-CSF) was allowed. Granulocyte colony-stimulating factor was used for the treatment of febrile neutropenia. Doses of CPT-11 and 5-FU were reduced by 15% in subsequent cycles in case of grade 3 or 4 diarrhoea. The 5-FU dose was reduced in case of grades 3–4 stomatitis or dermatitis. Oxaliplatin dose was reduced by 15% in cases of persistent (⩾14 days) paresthaesia or temporary (7–14 days) painful paresthaesia or functional impairment. In cases of persistent (⩾14 days) painful paresthaesia or functional impairment, L-OHP was omitted in subsequent cycles from the regimen until recovery.

### Patient evaluation

Pretreatment evaluation included a detailed medical history and physical examination, a complete blood cell count (CBC) with differential and platelet count, blood chemistry, serum levels of carcinoembryonic antigen and computed tomography scans of the chest and abdomen. Pretreatment evaluation had to be performed within 2 weeks prior to study entry. During treatment, a CBC with differential and platelets count was performed weekly and in case of grades 3–4 neutropenia or thrombocytopenia or febrile neutropenia it was performed daily until haematologic recovery. In addition, patients were clinically assessed and blood chemistry was performed before each treatment cycle. Response to treatment was evaluated every 2 months (four chemotherapy cycles) or sooner if clinically indicated.

The Response Evaluation Criteria in Solid Tumors (RECIST) were used to assess tumour responses (Therasse *et al*, 2000). Complete response required that all disease disappear without new lesions. Partial response required at least a 50% reduction in the sum of the products of the longest perpendicular diameters of all measurable lesions. Disease progression required 25% or greater increase in measurable tumour or an increase in tumour size in patients whose lesions did not meet the criteria for measurable disease. After partial response, tumour measurements exceeding 50% of the maximal extent of a previously observed reduction constituted progression. Any new lesion also constituted progression. Patients who did not meet the definitions of response or progression were classified as having stable disease.

The duration of response was measured from the first documentation of response to disease progression. The TTP was determined by the interval between the initiation of treatment and the date when disease progression was first documented or the date of death from any cause. Overall survival was measured from the date of treatment initiation to the date of death. The follow-up time was measured from the day of first treatment administration to the time of the present analysis (for patients still alive).

### Statistical considerations

**Figure 2:**
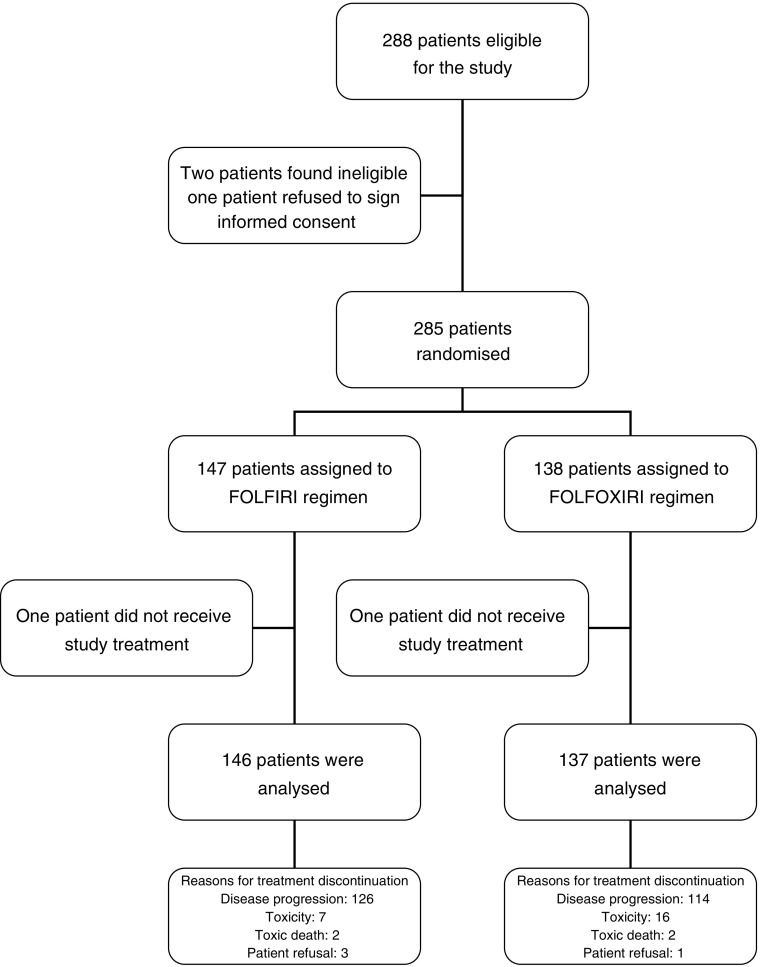
Trial profile.

**Figure 3:**
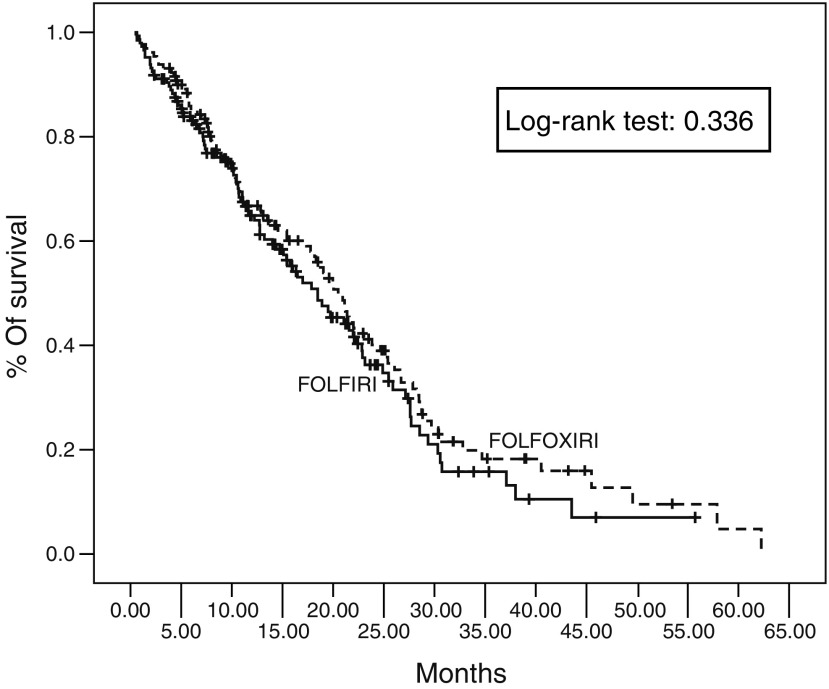
Kaplan–Meier overall survival of patients treated with FOLFIRI and FOLFOXIRI.

Randomisation was performed using a minimisation technique (Pocock and Simon, 1975), stratifying patients by centre, prior adjuvant chemotherapy (yes or not) and ECOG performance status (0–1 *vs* 2). The primary end point of the study was OS. Secondary end points were TTP, RR and tolerance. The study was designed for two sided log-rank test to have 80% power to detect a 25% improvement in survival for the experimental arm, based on the assumption that OS would be 17 months in the standard arm (FORFIRI) (Douillard *et al*, 2000) and 22.5 months for the experimental arm (FOLFOXIRI) (Souglakos *et al*, 2002) (type I error 5%, type II error 20%). Using the Freedman's formula, 136 patients per arm were required with the assumption that the accrual period would last 48 months (Freedman, 1982). An interim analysis using O’Brien–Fleming boundaries after 50% of patients progressed was planned (O’Brien and Fleming, 1979). The Kaplan–Meier method was used to estimate survival and PFS curves, and the log-rank test was used to compare the curves (Kaplan and Meier, 1958). Cox's proportional hazards modelling was used to calculate hazard ratios (HR) and confidence intervals (CIs;) (Cox, 1972). *χ*^2^ tests were used to compare toxicity and confirmed RR. *P-*values <0.05 were considered statistically significant for all comparisons. All randomised patients were included in an intention-to-treat analysis; patients who canceled before the initiation of therapy were excluded from toxicity analyses (Figures 2 and 3).

## RESULTS

### Patients characteristics

From October 2000 to December 2004, 285 patients (147 in arm A and 138 in arm B) with MCC were randomly assigned to receive front-line chemotherapy at 11 institutions and received at least one chemotherapy cycle. Two patients, one in each arm, received no study treatment because consents were withdrawn. Baseline characteristics of the remaining 283 patients are shown in Table 1. Almost half of the patients in both arms were ⩾65 years old; PS of 0–1 had 89% of patients in each arm; liver metastases were present in 70 and 72% of the arm A and B patients, respectively, whereas 16.4 and 24.6% of them, had ⩾3 involved sites. In 43 and 48% of the arm A and B patients, respectively, metastases were synchronous to diagnosis of the primary tumour. Overall approximately one-quarter of the patients in each arm were classified as high risk according to the Kohne prognostic index (Köhne *et al*, 2002).

### Efficacy

With a median follow-up period of 26 months (range, 1–62 months), 85% of patients had disease progression and 62% had died. The OS was not significantly different between the two arms (FOLFIRI arm: 19.5 months (range: 1.0–55.7)) and (FOLFOXIRI arm: 21.5 months (range 1–62.3)), (*P*=0.337). The probability of 1- and 2-years survival was 64 and 34% in arm A and 67 and 43% in arm B, respectively (Figure 1). Multivariate analysis revealed that independent prognostic factors for decreased survival were PS of 2 and nonresponse to treatment with HR 2.5 (95% CI: 1.701–3.703; *P*=0.0001) and 2.102 (95% CI: 1.598–2.765; *P*=0.0001), respectively. In contrast age, treatment arm and prior adjuvant chemotherapy was not significant factors for the patients’ outcome. In the FOLFIRI group the OS was 20 months for patients with PS 0–1 and 6.4 months for patients with PS of 2 (*P*=0.03); in the FOLFOXIRI group the OS was 24 moths for the patients with PS of 0–1 and 6.6 months for the patients with PS of 2 (*P*=0.0001). There was no statistical difference in terms of OS in young or aged patients irrespectively of the received regimen (FOLFIRI arm: <65 years: OS 19.9 months; ⩾65 years: OS 16.9 months; *P*=0.452; FOLFOXIRI arm: <65 years: OS 22.1 months; ⩾65 years: OS19.9 months; *P*=0.263). Patients enrolled in the FOLFIRI arm had a significantly better OS when received second-line chemotherapy (median OS 21 months; range: 15.9–55.7) compared to those who did not (median OS 12.2 months; range: 7.82–16.64) (*P*=0.016); this was not the case for patients who were enrolled to the FOLFOXIRI arm (median OS 23 months; range: 17.6–62.3 for those who received second-line chemotherapy) *vs* (median OS 19.5 months; range: 13.4–25.5 for those who did not received second-line chemotherapy) (*P*=0.942).

**Figure 4:**
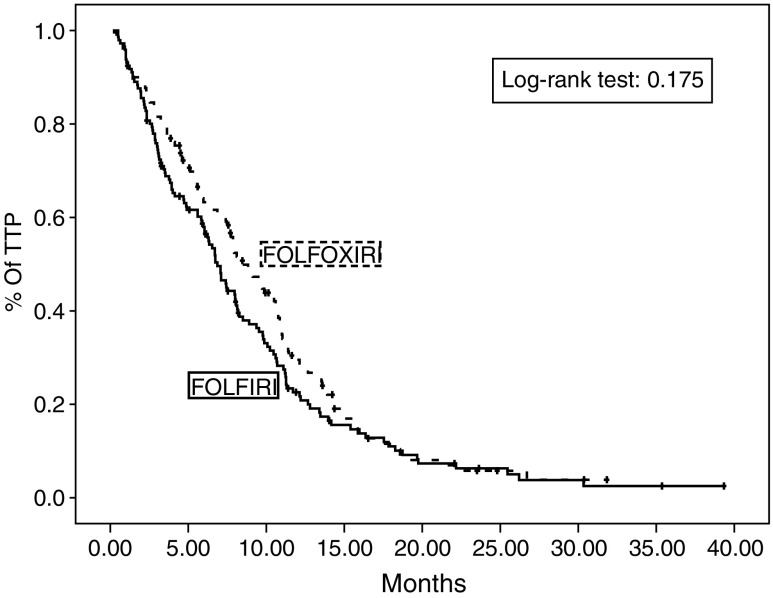
Kaplan–Meier time to tumour progression (TTP) of patients treated with FOLFIRI and FOLFOXIRI.

Median time to disease progression (TTP) was 6.9 months (95% CI: 6.0–7.7 months; range: 1.0–39.3) for patients receiving FOLFIRI and 8.4 months (95% CI: 7–10.2 months; range: 1.0–32.3) for patients receiving FOLFOXIRI with a HR of 0.83 (95% CI: 0.64–1.08; *P*=0.17) (Figure 4). In the FOLFIRI arm, TTP was 7.1 months (range: 1–39.3) for patients with PS of 0–1 and 2 months (range: 1–10.7) for patients with PS of 2 (*P*=0.0001); in the FOLFOXIRI arm, TTP was 9.7 (range: 1.0–32.3) and 4.1 (range: 1.0–15.9) months for patients with PS of 0–1 and 2, respectively (*P*=0.0047). In Cox's multivariate analysis PS of 2 (HR: 1.857, 95% CI: 1.217–2.834, *P*=0.004) and no response to treatment (HR: 2.166, 95% CI: 1.553–3.020, *P*=0.0001) but not age were emerged as independent prognostic factors for TTP. Although it was a trend for lower TTP prior adjuvant chemotherapy it was not a significant factor for patient's outcome. Age and treatment arm were not important prognostic factors for the TTP.

### Response to treatment

Five (3.4%) CRs were observed in the FOLFIRI arm and nine (6.5%) in the FOLFOXIRI arm; in addition 44 (30.2%) and 50 (36.5%) patients enrolled in the FOLFIRI and FOLFOXIRI arm, respectively, experienced a PR for an overall RR of 33.6% for FOLFIRI and 43% for FOLFOXIRI (*P*=0.168). In all, 39 (26.7%) patients treated with FOLFIRI and 43 (31.3%) patients treated with FOLFOXIRI presented stabilisation of the disease while 58 (39.7%) and 35 (25.5%) patients, respectively, progressed under treatment. The median time of response duration was 9 (range: 1–27.1) and 9.7 months (range, 1–34.6) (*P*=0.44) in the FOLFIRI and the FOLFOXIRI arm, respectively.

Secondary metastasectomy was performed in six (4%) patients treated with FOLFIRI and 14 (10%) patients treated with FOLFOXIRI (*P*=0.08). Six patients (three in each arm) underwent resection of lung metastases and 14 patients of liver lesions (three in the FOLFIRI and 11 in the FOLFOXIRI arm). R0 resection could be realised in all patients with lung lesions and in 11 patients with liver metastases (two in arm A and nine in arm B).

### Compliance with the treatment

A total of 1212 treatment cycles were administered in the FOLFIRI arm and 1179 in the FOLFOXIRI arm. The median number of cycles was nine (range 1–22) and 10 (range 1–20) per patient treated with the FOLFIRI and the FOLFOXIRI regimen, respectively.

A total of 101 (8.3%) chemotherapy courses were delayed in the FOLFIRI and 166 (14%) in the FOLFOXIRI arm (*P*=0.04); the median duration of delay was 4 days (range, 1–14) in each arm. The reasons of delay were haematologic (FOLFIRI: *n*=44, 4.0%; FOLFOXIRI: *n*=74, 6.0%), nonhaematologic (FOLFIRI: *n*=9, 1%; FOLFOXIRI: *n*=23, 2.0%) or both (FOLFIRI: *n*=4, 0.3%; FOLFOXIRI: *n*=14, 1%) toxicity; 54 (4.0%) courses in the FOLFIRI and 55 (5.0%) in the FOLFOXIRI arm were delayed because of reasons unrelated to disease or treatment (i.e. pending imaging studies for response evaluation). The median interval between cycles was 16 days in both treatment arms. Dose reduction was required in 40 (3.0%) cycles in the FOLFIRI arm and 87 (7.0%) cycles in the FOLFOXIRI arm (*P*=0.001). In all, 10 (7.0%) patients discontinued treatment in the FOLFIRI arm and 16 (12.0%) in the FOLFOXIRI arm (*P*=0.296). The reasons for treatment discontinuation were haematologic (FOLFIRI: *n*=22 cycles, 2.0%; FOLFOXIRI: *n*=40, 3.0%) and nonhaematologic (FOLFIRI: *n*=18 cycles, 1.4%; FOLFOXIRI: *n*=47, 4.0%) toxicity. The delivered relative dose intensity was 85% for CPT-11, 84% for L-OHP and 88% for 5-FU/LV of the protocol-planned doses in the FOLFOXIRI arm and 90% for CPT-11 and 92% for 5-FU/LV in the FOLFIRI arm.

### Toxicity

Patients treated with the FOLFOXIRI regimen had a significantly higher incidence of severe alopecia (*P*=0.0001), diarrhoea (*P*=0.001) and neurosensory disorders (*P*=0.001) compared with patients treated with FOLFIRI (Table 2). There was no difference in the incidence of severe (grade 3/4) haematological toxicity. There were two treatment-related deaths in the FOLFIRI arm and two in the FOLFOXIRI arm. All four treatment-related deaths were due to febrile neutropenia combined with diarrhoea. The death rates within the first 60 days of treatment were 2.7% (95% CI, 1.1–4.6%) for patients treated with the FOLFIRI regimen and 2.9% (95% CI, 1.3–5.3%) for those treated with the FOLFOXIRI regimen.

Patients with PS of 2 presented a significantly higher incidence of grade 3/4 neutropenia (*P*=0.001), diarrhoea (*P*=0.001), fatigue (*P*=0.0001) and febrile neutropenia (*P*=0.02) in comparison with patients with PS of 0–1 in both treatment arms. In addition patients older than 65 years showed a significantly higher incidence of grade 3/4 diarrhoea in comparison with younger patients in both treatment groups (*P*=0.005 and 0.017, for FOLFIRI and FOLFOXIRI arm, respectively). There was no difference in terms of grade 3/4 haematologic or nonhaematologic toxicity for patients who had previously received adjuvant chemotherapy or/and radiotherapy.

### Second-line treatment

Chemotherapy regimens administered after first-line therapy are shown in Table 3. Although second-line treatments were not specified by the protocol we requested that therapies administered after progression on protocol therapy must be reported. A higher proportion (70%) of patients treated with FOLFIRI received second-line treatment; the majority of them were treated with L-OHP-based chemotherapy (XELOX or FOLFOX regimen). Conversely, relatively fewer patients (58%) treated with front-line FOLFOXIRI received second-line treatment compared with those who were treated with FOLFIRI (*P*=0.041). A small proportion of these patients received second-line CPT-11 and Cetuximab.

## DISCUSSION

This is the first randomised study comparing the efficacy of the combination of all active drugs against CRC (CPT-11, L-OHP, 5-FU and LV) as first-line treatment of patients with MCC. Although the observed OS (median 21.5 months) for the FOLFOXIRI arm is one of the longest observed in a randomised multicentre trial using any combination regimen for first-line therapy, the difference could not reach statistical significance. In addition, there was no difference between the two arms concerning the secondary end points of the study which were the TTP and the RR, although there is a trend for longer TTP with the triple drug regimen (HR:0.83; *P*=0.17; Figure 4).

The median OS achieved with the FOLFOXIRI regimen was similar with that reported from our group in a previous phase II study (Souglakos *et al*, 2002) and therefore was used for the statistical design of this trial. On the other hand, the OS in the standard (FOLFIRI) arm was 19.5 months, 2.5 months more than that reported in the initial FOLFIRI randomised trial (Douillard *et al*, 2000), which was also used for the statistical design of our trial. In the present study, the median age and the proportion of patients’ ⩾65 years old were higher in comparison with that reported in the initial FOLFIRI trial (10). In addition, our study enrolled patients >75 years old, a practice which was relatively unusual in the previous randomised studies (Douillard *et al*, 2000; Tournigand *et al*, 2004). Both the subgroup and the multivariate analyses could not reveal any significant difference concerning both the median OS and TTP between the older and the younger patients. In addition, the proportion of patients that were treated with prior adjuvant chemotherapy was higher in our study (almost half of them) in comparison with other randomised trials with FOLFIRI (Douillard *et al*, 2000; Tournigand *et al*, 2004). Therefore, patients’ characteristics could not be the reason for the improved survival of patients treated with front-line FOLFIRI. Conversely, the most possible explanation could be the administration of second-line chemotherapy. Tournigand *et al* (2004) reported a median OS of 21.5 months for patients treated with FOLFIRI followed by second-line FOLFOX6 which was comparable with the median OS of 21 months for the patients treated with FOLFIRI in the present study. The proportion of patients treated with second-line FOLFOX6 in the previous study (Tournigand *et al*, 2004) was similar with the proportion of patients treated with L-OHP-based chemotherapy (FOLFOX4 or XELOX) in the present study (74 and 63%, respectively). Overall the sequential treatment with FOLFIRI and FOLFOX (or the opposite) should be considered as the preferred treatment option, especially for the addition of molecular targeted therapy. An alternative choice could be the combination of CPT-11 and L-OHP without 5-FU, especially for patients that can not tolerate treatment with infusional 5-FU (Goldberg *et al*, 2004).

In a recent meta-analysis, of seven phase III trials in advanced CRC, it was shown that the median OS was significantly correlated with the proportion of patients receiving all active agents over the disease course but not with the proportion of patients receiving second-line chemotherapy (Grothey *et al*, 2004). The results of the present study seem to support this conclusion since the median OS for the patients who did not receive second-line chemotherapy after FOLFIRI failure was significantly lower compared with that of patients who were treated with L-OHP-based second-line chemotherapy (12.5 *vs* 21 months, *P*=0.016). In contrast, the median OS did not differ in FOLFOXIRI arm for patients treated or not with second-line chemotherapy (19.5 *vs* 23 months, *P*=0.942). Taking together these observations seem to indicate that patients who were exposed to all three drugs presented a better median OS (>20 months). Moreover, the survival of patients receiving all active drugs upfront (FOLFOXIRI patients) was similar with that of patients receiving the active drugs in sequence (i.e. FOLOFIRI first and L-OHP-based regimen on progression). Conversely patients who did not receive L-OHP during the evolution of the disease had significantly lower median OS. However, we cannot exclude that patients who lived longer had a better chance of receiving all therapies and all available active drugs, while patients with poorer and shorter life expectancy had a lesser chance to receive second-line chemotherapy.

Another important finding of the present study was that patients with PS of 2 presented a significantly lower median OS and TTP, irrespectively of the used chemotherapy regimen. In addition, in the multivariate analysis, PS of 2 was emerged as an independent prognostic factor for disease progression and OS. Furthermore, patients with PS of 2 experienced a significantly higher incidence of neutropenia, febrile neutropenia, anaemia, diarrhoea and fatigue in comparison with patients with a PS of 0–1 in both treatment arms. Based on these data, it could be proposed that patients with a PS of 2 would not be candidates for aggressive combination chemotherapy. In contrast, patients ⩾65 years did not differ in any examined parameter except the significantly higher incidence of diarrhoea complicating both regimens compared with younger patients. These findings are in agreement with two recently published phase II trials (Sastre *et al*, 2005; Souglakos *et al*, 2005) and strongly suggest that age alone should not compromise the treatment decision in elderly patients with advanced CRC. In addition, prior adjuvant chemotherapy has not any significant effect in the patient's outcome (Table 2).

As expected, the FOLFOXIRI regimen had a less favourable toxicity profile than FOLFIRI regimen. Patients treated with FOLFOXIRI had a significantly higher incidence of severe alopecia, diarrhoea and neurosensory toxicities compared with patients treated with FOLFIRI; however, the death rates within the first 60 days of treatment and the proportion of toxic deaths were the same in both arms. Despite the observation that the proportion of patients who discontinued treatment was similar in the FOLFIRI (7%) and the FOLFOXIRI (12%) arms, dose reductions and treatments delays were significantly more frequent in the FOLFOXIRI arm resulting in lower dose intensity rates.

In conclusion, the present study failed to demonstrate any survival advantage of the FOLFOXIRI over the FOLFIRI regimen. The improved OS of patients treated with front-line FOLFIRI and especially for those who received second-line L-OHP-based chemotherapy, emphasises the importance to treat with all the available chemotherapeutic drugs (CPT-11, L-OHP, 5-FU) patients with advanced CRC who are candidates for this type of therapy.

## Figures and Tables

**Table 1:** Patients characteristics

	**FOLFIRI (*n*=146)**		**FOLFOXIRI (*n*=137)**	
**Characteristics**	***n***	**%**	***n***	**%**
*Age*				
Median (range)	66 (39–84)		66 (25–82)	
⩾65 years	82	56	75	55
				
*Sex*				
Male	82	58	76	55
Female	61	42	61	45
				
*Performance status (ECOG)*				
0	55	38	49	36
1	74	51	73	53
2	17	11	15	11
				
*Localisation*				
Colon	110	75	100	73
Rectum	36	25	37	27
				
*Sites of disease*				
Liver	102	70	99	72
Lung	46	32	42	31
Other	63	43	61	46
				
*Number of metastatic sites*				
1	59	40	55	40
2	63	43	57	42
⩾3	24	16	25	18
Median (range)	2 (1–4)		2 (1–6)	
				
*Metastases*				
Synchronous	63	43	66	48
Metachronous	83	57	70	52
				
*Kohne prognostic index*				
Low risk	54	37	44	32
Intermediate risk	57	39	56	41
High risk	35	24	37	27
				
*Prior therapy*				
Adjuvant chemotherapy	48	33	49	36
Adjuvant		18		17
Chemoradiotherapy		12		12

**Table 2:** Incidence of common toxicities with the FOLFIRI and FOLFOXIRI regimens (maximum toxicity per patient)

	**FOLFIRI (146)**	**FOLFOXIRI (130)**		**FOLFIRI (146)**	**FOLFOXIRI (130)**	
	**Any**			**Grade 3/4**		
	**%**	**%**	***P*-value**	**%**	**%**	***P*-value**
Neutropenia	60	73	NS^a^	28	35	0.192
Febrile neutropenia	6	9	NS	4	7	0.186
Thrombocytopenia	20	31	NS	4	2	0.4
Anaemia	59	60	NS	1	4	0.072
Nause/vomiting	45	52	NS	4.8	4.6	0.944
Diarrhoea	51	69	NS	10.9	27.7	0.0001
Mucocitis	18	21	NS	4	5	0.748
Neurological	11	59	0.001	0	5.8	0.001
Cutaneous	15	21	NS	3	4	0.133
Alopecia	56	74	NS	12	32	0.0001
Fatigue	36	41	NS	5	5.6	0.944

a NS, nonsignificant.

**Table 3:** Second-line therapies

	**FOLFIRI**		**FOLFOXIRI**		
**Second-line treatment**	**No patients**	**%**	**No patients**	**%**	***P*-value**
Any	102	70	80	58	0.041
L-OHP based	92	63	39	28	0.029
CPT-11 based	10	6	14	10	NS^a^
Fluoropyrimidines	44	30	29	21	NS
Cetuximab	10	7	7	5	NS

a NS, nonsignificant.
